# Hotspots and frontiers in chronic postoperative pain: a bibliometric analysis and review (2004–2025)

**DOI:** 10.3389/fmed.2026.1730431

**Published:** 2026-03-03

**Authors:** Weijuan Wang, Lingqin Zhou, Lingling Ren, Guanglan Chen, Xiangcheng Hu

**Affiliations:** Digestive Endoscopy Center, The Second People's Hospital of Lishui, Lishui, Zhejiang, China

**Keywords:** bibliometrics, chronic postoperative pain, hotspots, pathogenesis, review

## Abstract

**Background:**

Surgery is a common cause of chronic pain. Current treatments for postoperative chronic pain yield suboptimal outcomes, with slow clinical progress.

**Methods:**

This study employed bibliometric analysis of chronic postoperative pain (CPSP)-related literature published between January 1, 2004, and August 5, 2025, using the Web of Science and Scopus databases. A total of 1,211 eligible English articles and reviews were screened, analyzing publication trends, countries/regions, institutions, authors, journals, and keywords.

**Results:**

From 2004 to 2025, annual publications in CPSP showed an upward trend, with the United States, China, Canada, and Denmark being the top publishing countries. Keyword analysis revealed “risk factor,” “chronic postoperative pain,” and “pain management” as research hotspots. Highly cited authors and journals were predominantly concentrated in North America and Europe.

**Conclusion:**

CPSP research is expanding, yet effective treatment options remain scarce. Future studies should enhance multidisciplinary collaboration to deepen understanding of CPSP pathogenesis and develop more effective interventions.

## 1 Introduction

Despite more than 30 years of research, approximately 312 million surgeries are still performed worldwide each year (1). Surgery is a common and significant contributor to chronic pain (2). Chronic pain refers to pain that lasts or recurs for more than 3 months (3). Research shows that in the United States alone, more than 50 million Americans suffer from chronic pain each year, with approximately 40% experiencing activity limitations due to chronic pain. The resulting annual medical costs and economic burden of lost productivity exceed $560 billion (4, 5). Patients with CPSP often experience neuropathic pain (NP) symptoms such as hyperalgesia, allodynia, and paresthesia. Those with concomitant neuropathic pain typically exhibit more severe pain, greater limitations in daily activities, and a more pronounced impact on quality of life (6). Surgical-induced nerve injury triggers peripheral and central sensitization. Through mechanisms such as abnormal discharge in damaged sensory axons, ion channel remodeling, and altered gene expression in dorsal root ganglia, acute postoperative pain is transformed into a chronic pain state (7). Furthermore, research indicates that the increase in chronic pain incidence is associated with increased exposure to prescription opioids and opioid use disorders and overdose in some individuals (8). However, the vast majority of individuals who use prescription opioids do not abuse these medications. Nevertheless, opioids can induce feelings of euphoria, relaxation, and satisfaction, leading many patients to develop excessive dependence on such drugs, while others may experience misuse and drug-dependent addiction (9). Additionally, significant variations exist in postoperative pain and opioid use. For instance, across four common surgeries (laparoscopic cholecystectomy, hernia repair, hip replacement, knee replacement) and 103 hospitals, postoperative pain was widespread and highly variable (ranging from 0 to 9 on a 10-point scale) (10). Among nearly 2,400 patients across 12 procedures, the interquartile range variation in postoperative opioid consumption was 25-fold (11). Following cesarean sections, many patients took no more than 5 opioid pills, while approximately 20% took all or nearly all of the roughly 30 pills prescribed at discharge (12, 13). After thoracic surgeries, nearly half of patients took at least five pills, and nearly 30% took all or nearly all of their prescribed supply (13). Among children, postoperative opioid prescriptions also varied significantly following routine outpatient tonsillectomies and hernia repairs (14). This pattern was observed across many other procedures, both in adults and children (15, 16). These phenomena suggest that current understanding of chronic pain is insufficient, leading to slow progress in clinical applications. Therefore, it is essential to understand the current state of chronic pain to better address future challenges, particularly in the context of chronic postoperative pain.

Bibliometrics is the quantitative and qualitative analysis of literature in a specific field of research (17). Specifically, it assesses the contribution and impact of a series of indicators, including publication output, authors, countries/regions, institutions, journals, and keywords, with the aim of studying the current state, trends, and cutting-edge developments in the field (18, 19).

Therefore, it is very important to conduct a bibliometric study of the field of chronic postoperative pain (CPSP)research. Through this analysis, it is possible to identify the focus and emerging directions of research in this field.

## 2 Materials and methods

### 2.1 Database and search strategy

We selected the Web of Science Core Collection (WOSCC) database and Scopus database as the search database. The main search term was “chronic postoperative pain,” and the search was refined using information from previous studies. The search formula utilized in Web of Science is as follows: TS = “chronic postoperative pain” or “chronic postsurgical pain”. Language: English. Publication time: 2004.01.01–2025.08.05. The search formula utilized in Scopus is as follows: (TITLE-ABS-KEY ({chronic postoperative pain}) OR TITLE-ABS-KEY ({chronic postsurgical pain})) AND PUBYEAR >2004 AND PUBYEAR <2025 AND (LIMIT-TO (DOCTYPE, “ar”) OR LIMIT-TO (DOCTYPE, “re”)) AND (LIMIT-TO (LANGUAGE, “English”)).

We conducted a thorough search and screening process to minimize potential biases caused by database updates. We also implemented strict measures to address potential duplicate publications. We used EndNote X9 to remove duplicates from data directly exported from the WOSCC database and Scopus database. The software detects and removes duplicates by matching details such as titles, authors, publication dates, DOIs, and journal names. The software ensures that no duplicates or search strategies are present when inputting the database into the analysis. Three researchers then manually reviewed the literature after duplicate removal to ensure no duplicates were left behind. They checked titles, abstracts, and full texts.

### 2.2 Data analysis

We created flowcharts in Microsoft Word 2019 and statistical tables and curve fitting analyses in Microsoft Excel 2019. We used the bibliometrix 4.1.3 tool in R 4.3.1 software to perform Lotka law analysis and Bradford law analysis. Through the online bibliometrics website^1^, we were able to visualize international cooperation between countries. Additionally, bibliometric analysis of institutions, authors, journals, and keywords was conducted using VOSviewer 1.6.19 and Citespace 6.2R4. The primary focus was on examining co-author, co-occurrence, and co-citation patterns. Overlapping items were merged into a single unified element, spelling errors were manually corrected, and data cleaning was performed prior to extracting the data for follow-up analysis.

### 2.3 Lotka’s law and Bradford’s law

Lotka’s law is used to highlight the relationship between author productivity and publication frequency (20). This law can be used to assess inequality or concentration in a field, as well as to quantify author productivity. Authors with a single publication far outnumber those with a large number of publications, which can be used to identify influential authors in a field of study. Lotka’s law can be expressed in mathematical terms as follows (21):

$$A(n)=A(1)/n^{2}$$

In this equation, A(*n*) represents the total number of papers published by *n* authors, while A(1) corresponds to the total number of articles written by a single author.

Bradford’s Law can be used to divide journals into three different zones. Zone 1 is the core zone, covering the vast majority of journals. Zone 2 covers approximately 25% of journals. The third zone only includes journals that have published a few articles on the subject (22).

## 3 Result

**Figure 1:**
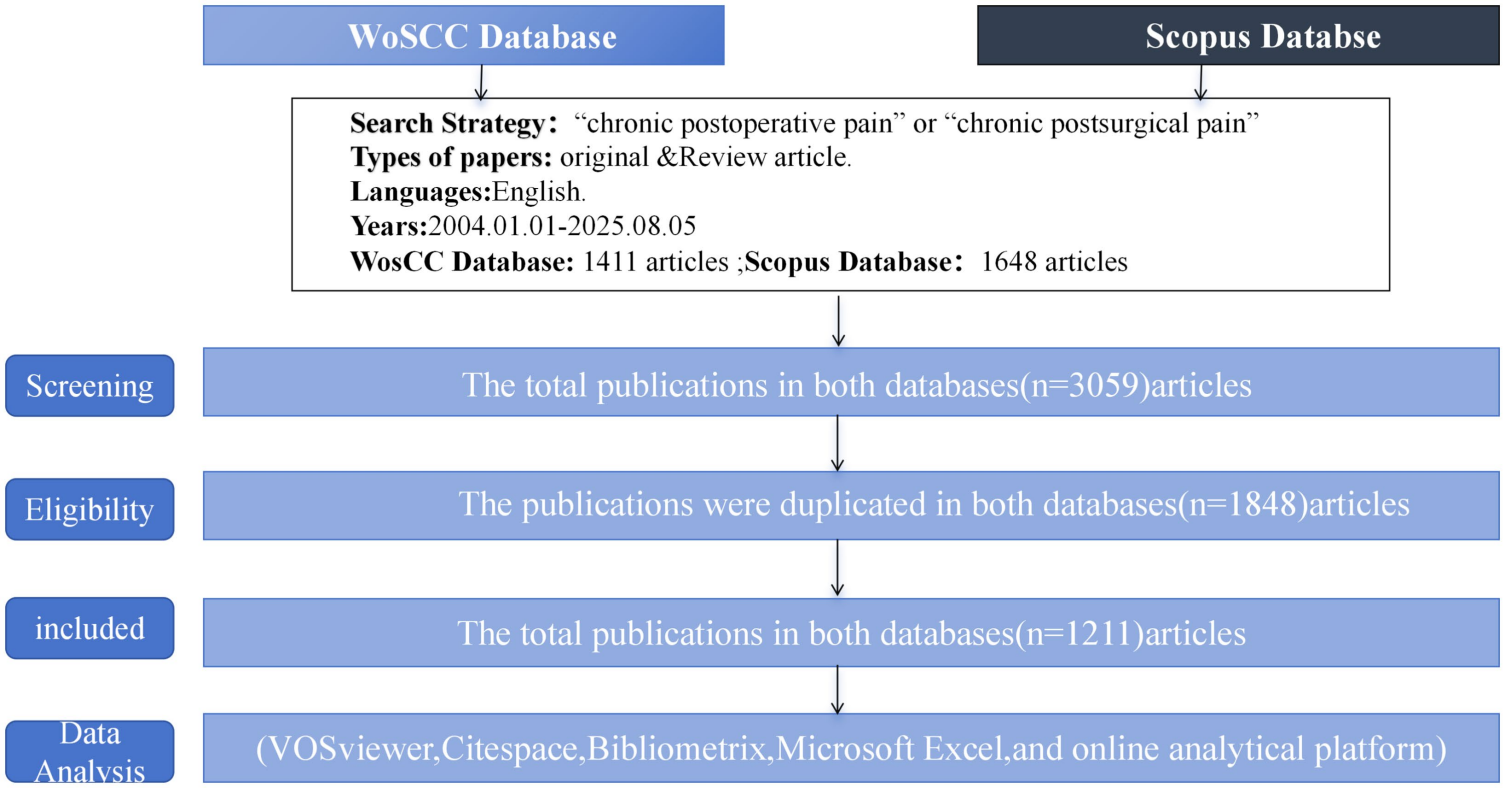
Flowchart illustrating the literature screening process (PRISMA flow diagram).

Initially, 3,059 articles were retrieved; however, two independent researchers (Zhou Lingqin and Ren Lingling) subsequently screened each article for relevance. During the literature screening process, any disagreements between the two researchers were resolved by a third researcher (Chen Guanglan), who made the final decision. After excluding irrelevant studies, 1,211 articles related to CPSP were ultimately selected for the bibliometric analysis. The detailed literature screening process is illustrated in Figure 1.

### 3.1 Analysis of the number of publications

**Figure 2:**
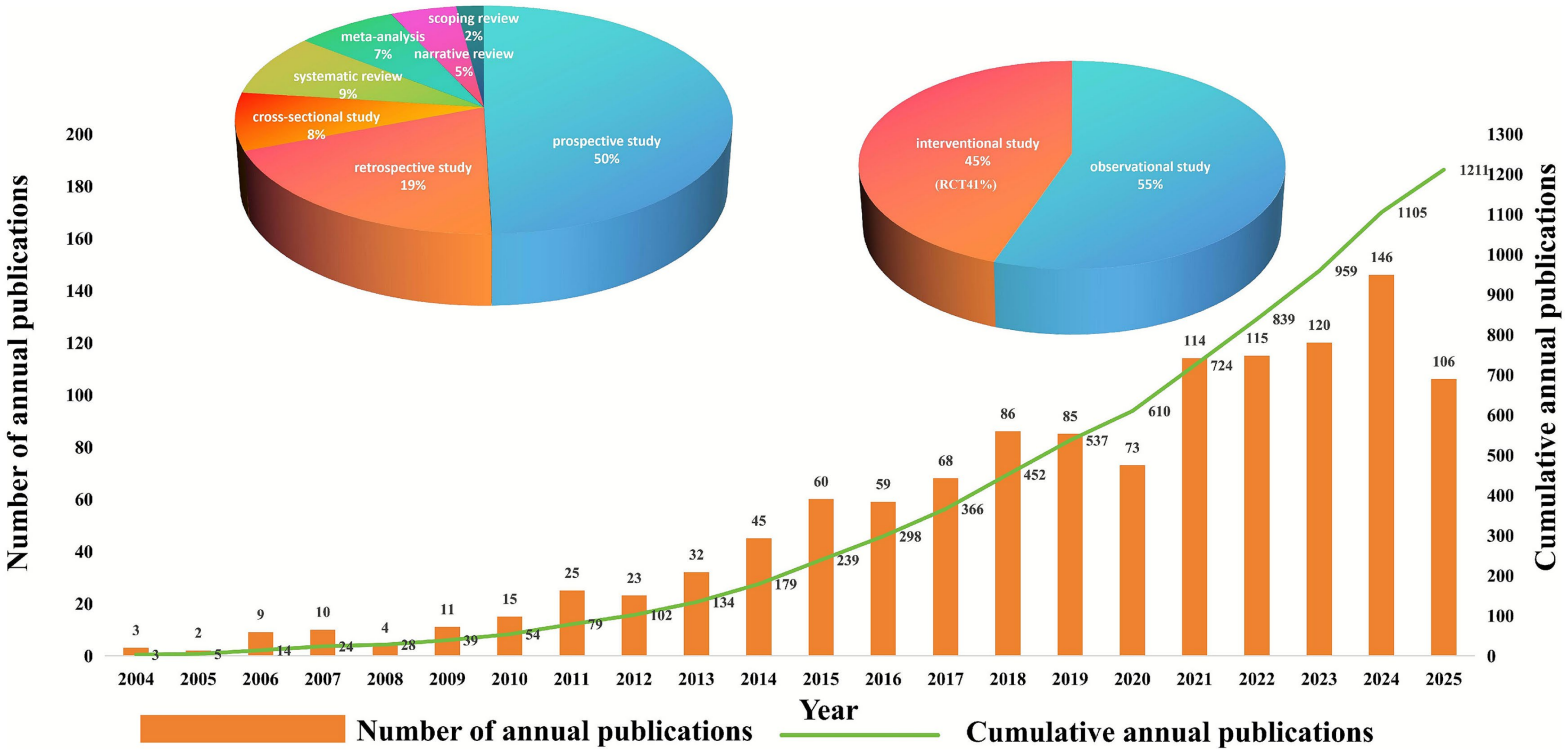
Trend graph depicting the annual growth in publications on CPSP.

The overall growth trend in the annual number of articles published in the field of CPSP is shown in Figure 2. As illustrated, only 3 relevant publications appeared in 2004, followed by a gradual increase reaching 15 articles by 2010. Subsequently, the number of publications demonstrated significant growth, reaching 60 in 2015 and further increasing to 73 in 2020. In 2024, the annual publication count reached 146 articles, with projections indicating continued upward momentum in 2025. The line graph depicts cumulative publications from 2004 to 2025. The cumulative total stood at 3 articles in 2004, rising to 54 by 2010. The cumulative count has steadily increased over time, reaching 610 in 2020, 1,105 in 2024, and is projected to reach 1,211 in 2025. Additionally, the pie chart illustrates the distribution of publication types. Among the total 1,211 publications, original research articles accounted for 77%, including 8% cross-sectional studies, 50% prospective studies, and 19% retrospective studies. Review articles accounted for 23% of the total, including 9% systematic reviews, 7% meta-analyses, 5% narrative reviews, and 2% scoping reviews. Among these, RCTs accounted for 41%, indicating a strong emphasis on high-quality evidence in this field. Prospective studies collectively constituted 50%, with cohort designs being the predominant type of observational research. Systematic reviews and meta-analyses totaled 193 articles, representing 16% of all reviews, reflecting a significant demand for knowledge synthesis and evidence-based practice.

### 3.2 Situation of countries/regions and institutions

**Figure 3:**
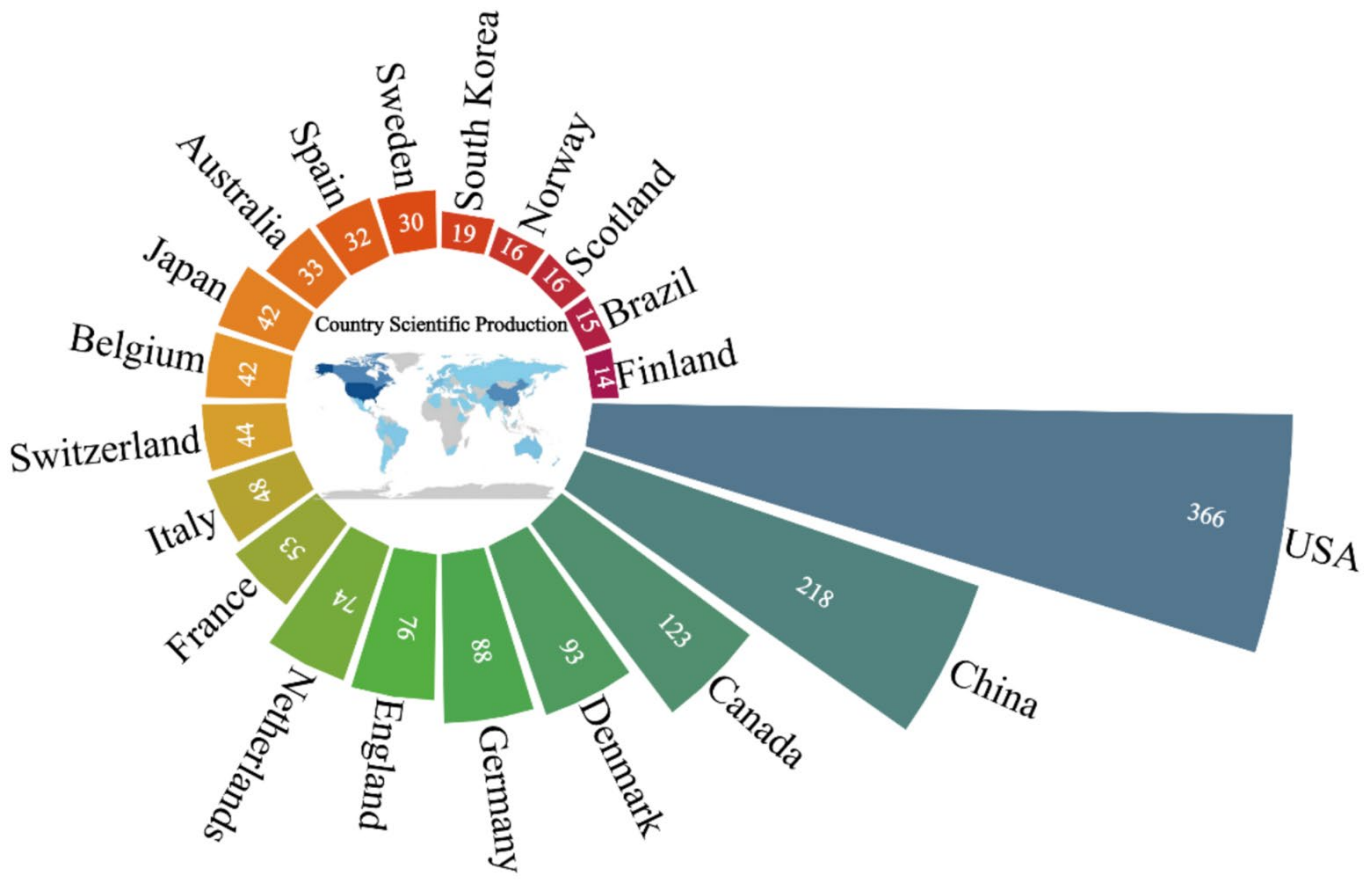
Nightingale’s rose chart of the top 20 countries by publications.

**Figure 4:**
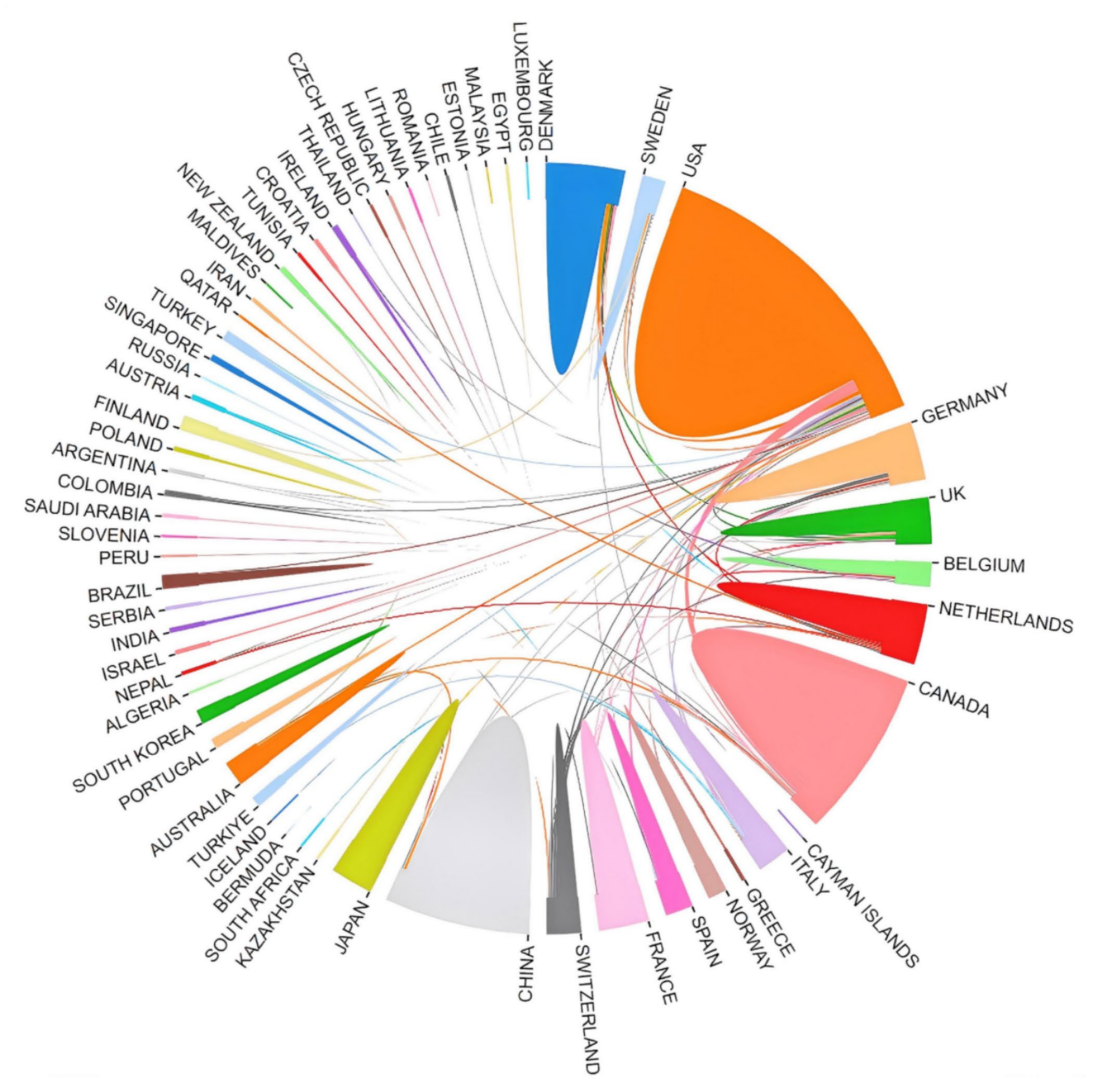
Network of cooperation between countries.

A total of 69 countries published articles on CPSP. As shown in Figure 3, the top 20 countries by productivity are listed, clearly illustrating each country’s scientific production in the field of CPSP. The United States, China, Canada, and Denmark exhibit the most significant output in this field, occupying the top four sectors of the rose diagram and demonstrating high levels of scientific productivity. The United States leads with 366 publications, underscoring its dominant position in this area. China (218 publications), Canada (123 publications), and Denmark (93 publications) follow closely, indicating substantial research activity in these three countries. Figure 4 reveals collaborative relationships among different countries. It shows that countries such as China, the United States, Canada, and Denmark play significant roles in international cooperation within the field of CPSP. Close collaborative ties exist between these countries, indicating robust cooperative networks. Particularly notable is the strong collaborative connection between China and the United States, potentially reflecting complementary research strengths and cooperative potential between the two countries in this field.

**Figure 5:**
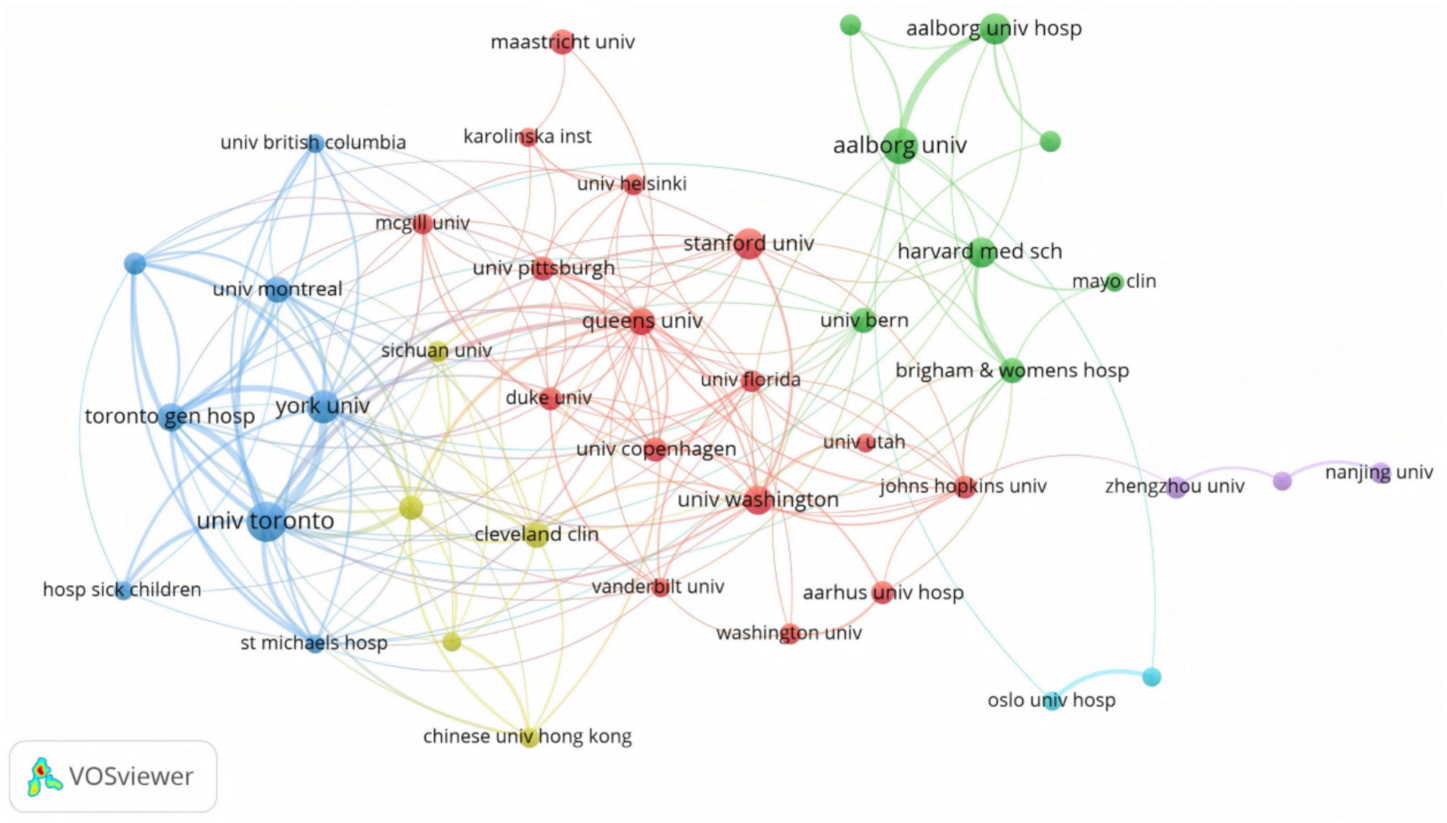
A network visualization map of institutions in the field of CPSP.

Figure 5 illustrates the collaborative network among different research institutions in the field of CPSP. The diagram reveals that institutions such as the University of Toronto, Toronto General Hospital, Aalborg university, Queen’s University, and Harvard Medical School occupy central positions within the network. This highlights their significant influence and extensive collaborative networks within the field. These institutions not only feature larger node sizes but also maintain a higher number of connections with other institutions, indicating their pivotal roles in research within this field. Table 1 provides the total number of articles, total citations, and average citations per article for the top 20 institutions. These metrics serve as key indicators for measuring institutional research impact and academic contributions. The University of Toronto leads with 142 articles, demonstrating high research output in the field of CPSP. Toronto General Hospital tops the list with 734 citations, indicating its research enjoys high recognition and influence within the academic community. Furthermore, Aarhus University Hospital’s average citation counts of 19.93 significantly exceeds that of other institutions, further confirming its leading position in this field. In departments studying chronic postoperative pain (Supplementary Table S1), we found orthopedic and general surgery to be the fields with the highest concentration of such research. This is particularly notable for amputation procedures and inguinal hernia repairs, likely due to their high surgical volumes and elevated incidence of postoperative pain. Additionally, research in thoracic and breast surgery has rapidly expanded, with open chest surgeries and breast surgeries emerging as key research topics over the past few years.

**Table 1:** The top 20 leading institutions in CPSP research.

Rank	Institution title	Records	Total citations	Average citation
1	University of Toronto	142	1,561	10.99
2	Aalborg University	100	668	6.68
3	McMaster University	65	432	6.65
4	Stanford University	62	131	2.11
5	Aalborg University Hospital	61	371	6.08
6	Queens University	52	885	17.02
7	University of Florida	50	207	4.14
8	Harvard Medical School	47	183	3.89
9	University of Washington	43	253	5.88
10	York University	42	697	16.6
11	Cincinnati Children’s Hospital Medical Center	41	453	11.05
12	Toronto General Hospital	38	734	19.32
13	University of Pittsburgh	37	78	2.11
14	University of Bern	36	407	11.31
15	Université de Montréal	33	253	7.67
16	Maastricht University	32	406	12.69
17	Sichuan University	31	66	2.13
18	Cleveland Clinic	28	184	6.57
19	Aarhus University Hospital	27	538	19.93
20	Radboud University Nijmegen	27	66	2.44

### 3.3 Authors analysis

**Figure 6:**
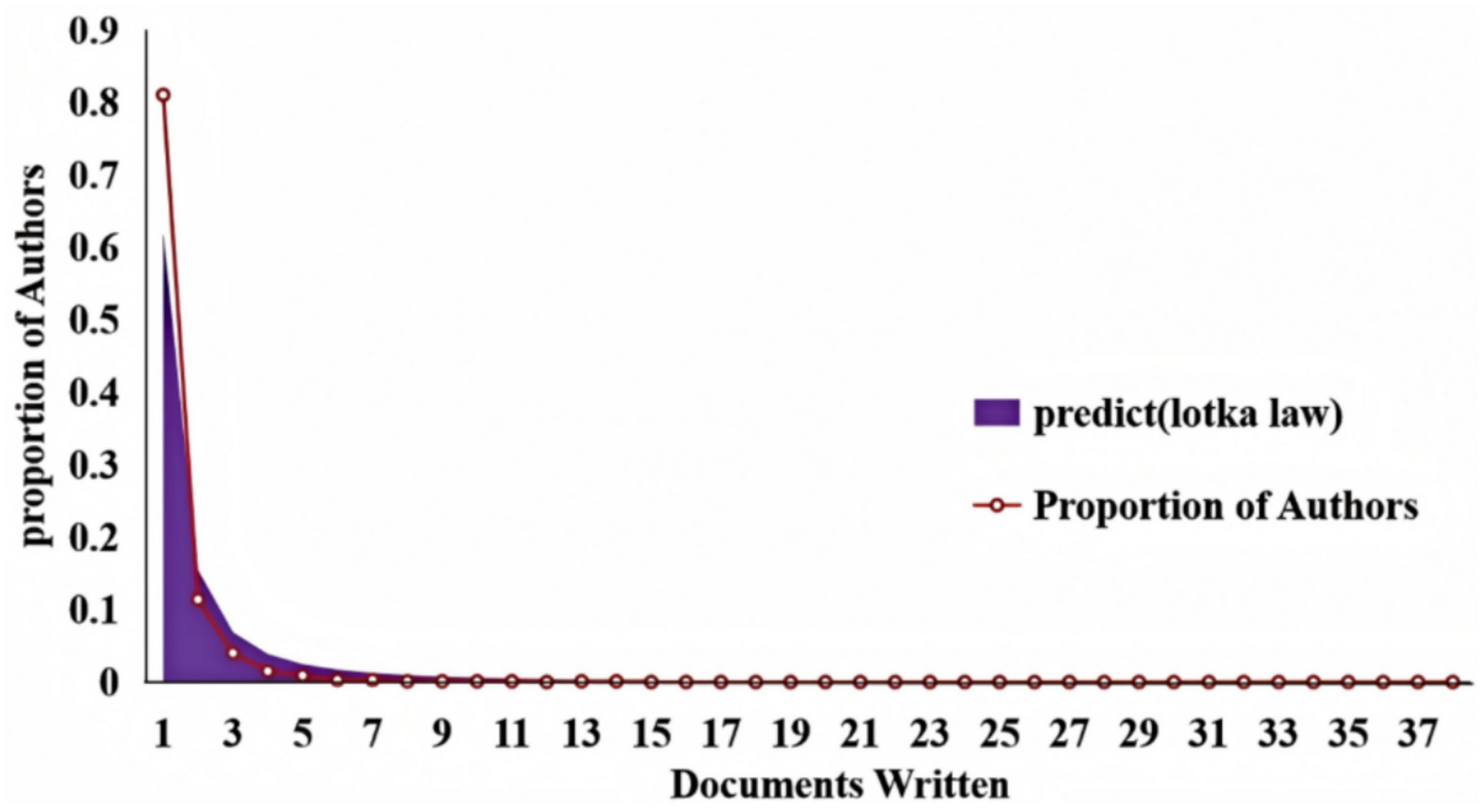
Scientific productivity of authors based on Lotka’s law.

**Figure 7:**
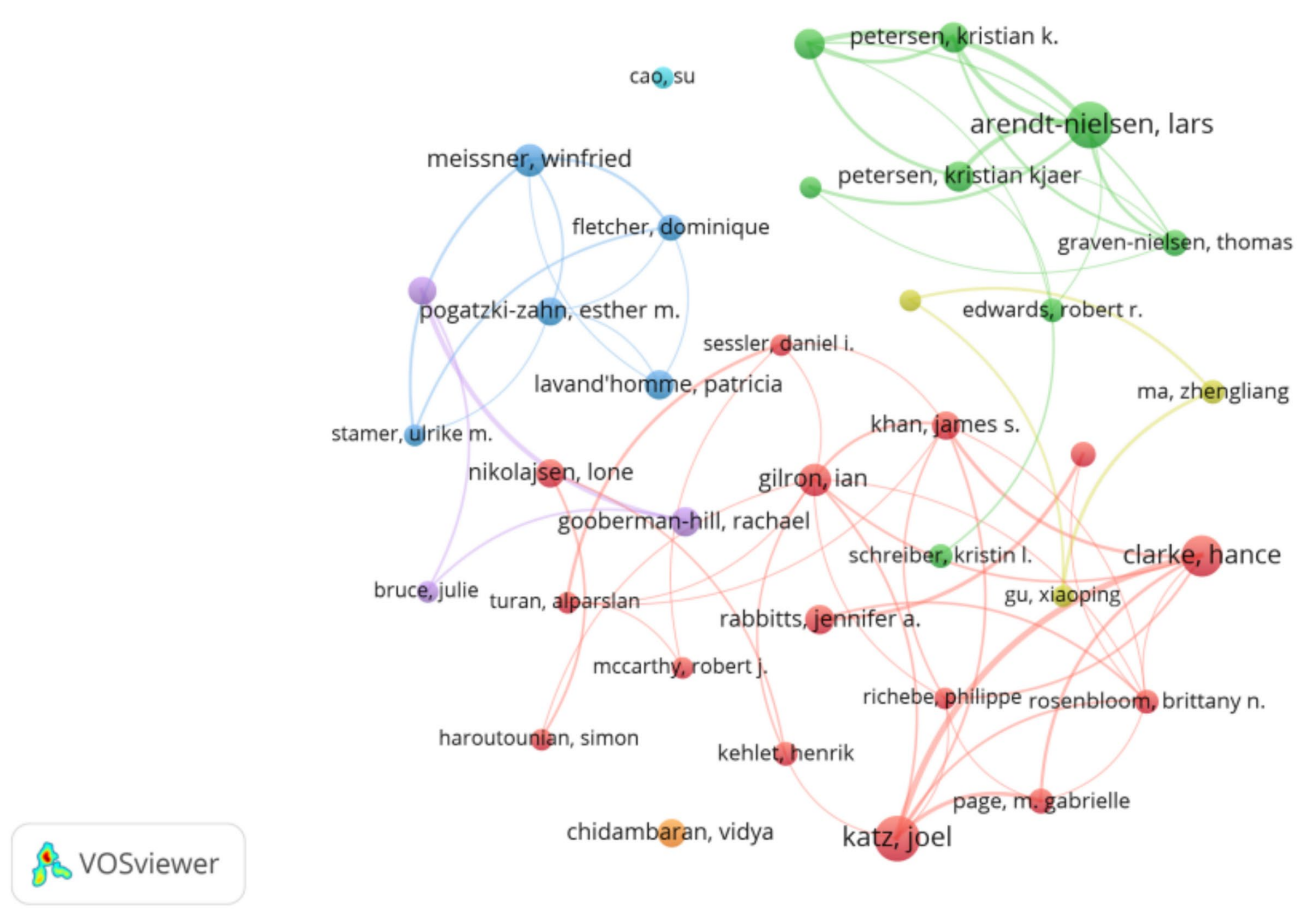
Network diagram of author collaborations for CPSP studies.

A total of 5,727 authors participated in research on CPSP. Analysis of scientific productivity using Lotka’s Law indicates that, as shown in Figure 6, the majority of authors (nearly 0.8) produced only one document. Table 2 provides details on the top 20 most productive and most cited authors in this field. By analyzing this data, we can more precisely assess each author’s academic influence. Arendt-Nielsen, L leads with 38 articles and an average citation count of 16.72, demonstrating high productivity within the field. Katz, J ranks second with 36 articles and 602 citations, also averaging 16.72 citations per paper, indicating significant influence. Kehlet, H, despite publishing only 13 articles, achieved a total of 309 citations, resulting in an average citation count of 23.77. This indicates exceptionally high average impact per article. Nikolajsen, L possesses the highest average citation count at 20.69. Although he authored only 13 articles, each carries significant influence. Figure 7 illustrates the collaborative relationships among authors in this field. The figure reveals that Katz, Joel and Clarke, Hance are central authors within the collaboration network, as indicated by their larger nodes signifying higher scientific output. Additionally, Gilron, Ian and Nikolajsen, Lone demonstrate high collaboration frequency, highlighting their significant influence within the field.

**Table 2:** The top 20 most prolific and cited authors in the field of CPSP.

Rank	Authors	Counts	Total citations	Average citation
1	Arendt-Nielsen, L	38	380	10
2	Katz, J	36	602	16.72
3	Clarke, H	29	256	8.83
4	Petersen, KK	28	342	12.21
5	Meissner, W	17	207	12.18
6	Gilron, I	17	273	16.06
7	Gooberman-Hill, R	15	119	7.93
8	Edwards, RR	14	57	4.07
9	Wylde, V	14	118	8.43
10	Simonsen, O	14	228	16.29
11	Nikolajsen, L	13	269	20.69
12	Rabbitts, JA	13	129	9.92
13	Kehlet, H	13	309	23.77
14	Lavand’homme, P	12	121	10.08
15	Chidambaran, V	12	99	8.25
16	Wang, J	12	11	0.92
17	Liu, Y	11	10	0.91
18	Pogatzki-Zahn, EM	11	25	2.27
19	Khan, JS	11	74	6.73
20	Graven-Nielsen, T	10	117	11.7

### 3.4 Journals analysis

**Figure 8:**
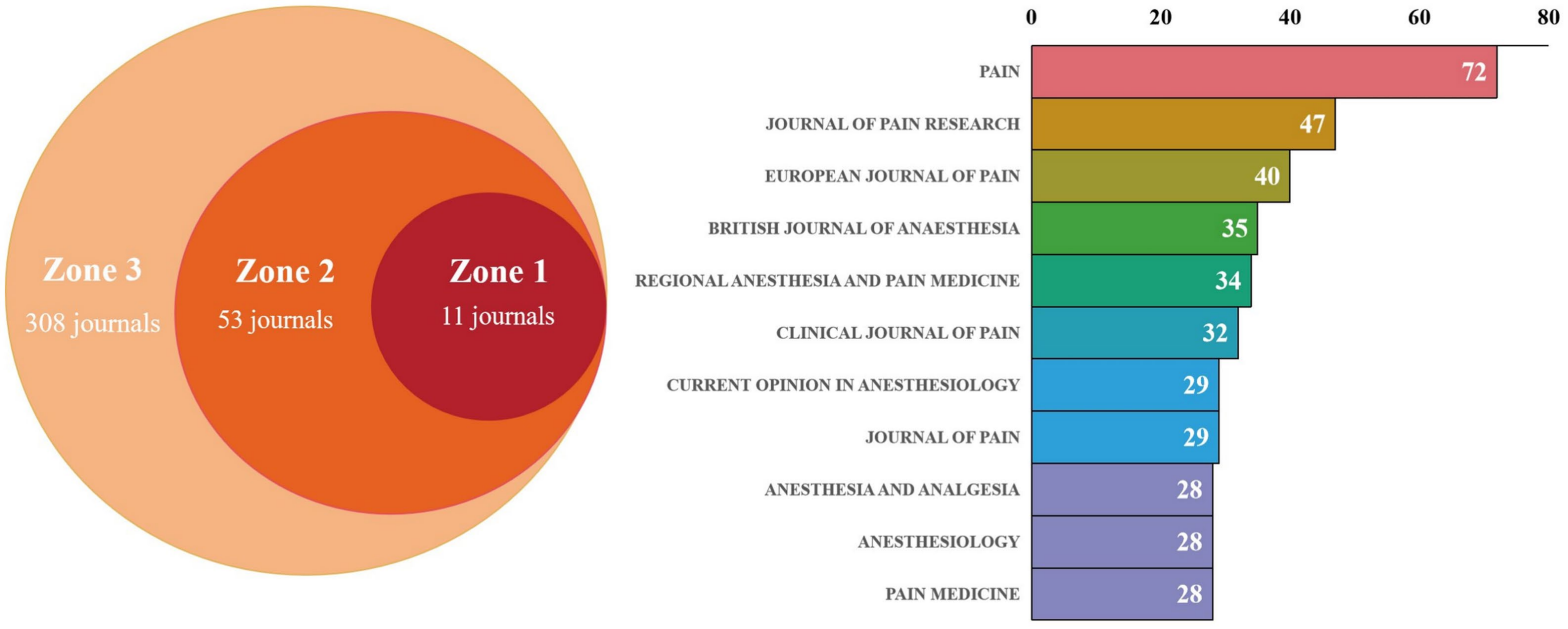
Scientific productivity of journals based on Bradford’s law and distribution map of journal publications in zone one.

**Figure 9:**
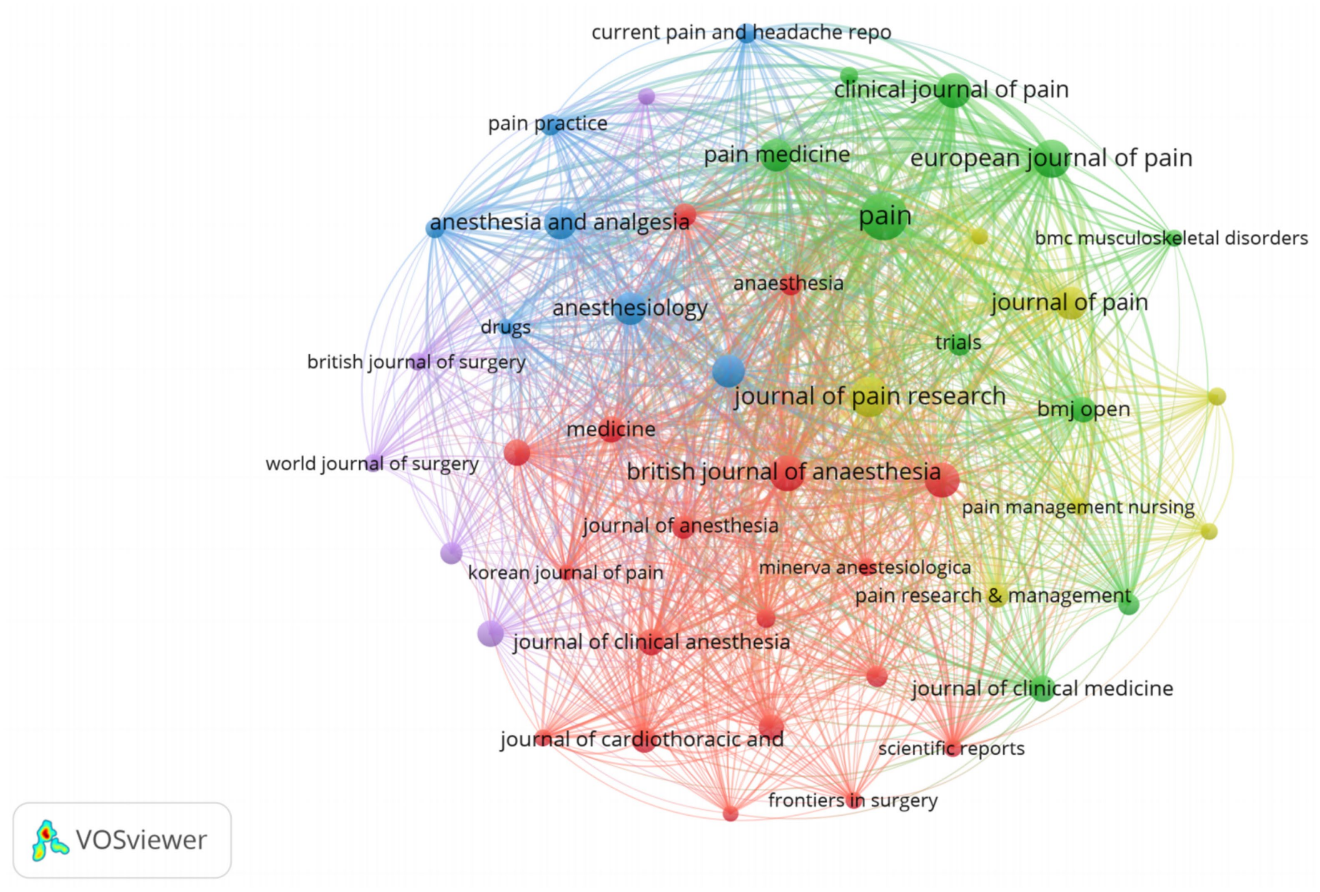
The network diagram of cited journals.

Bradford’s law has been applied to the evaluation of CPSP in core journals (23). As shown in Figure 8, the journal scientific output map categorizes journals into three zones: Zone 1 (Core Zone, 11 journals), Zone 2 (Secondary Core Zone, 53 journals), and Zone 3 (Peripheral Zone, 308 journals). The core zone contains the fewest journals but generates the highest scientific output, indicating these publications possess significant influence and concentration within their fields. Table 3 lists metrics for the 11 Core Zone journals, including publication volume, total citations, average citation count, and impact factor. The table shows that the journal “PAIN” stands out in terms of total articles, total citations, and average citations per article, while also possessing a high impact factor. This indicates the journal’s significant academic influence in the field of CPSP. Following closely are “JOURNAL OF PAIN RESEARCH,” “EUROPEAN JOURNAL OF PAIN,” and “BRITISH JOURNAL OF ANESTHESIA,” establishing themselves as the most representative journals in this field. Figure 9 illustrates the collaborative network among these journals. Core journals such as “PAIN”, “JOURNAL OF PAIN RESEARCH” and “EUROPEAN JOURNAL OF PAIN” occupy central positions in the network, indicating high collaboration frequency and influence within the field.

**Table 3:** The top 11 issued and cited journals for CPSP studies base zone one.

Rank	Journal title	Records	Total citations	Average citation	JCR (2025)	IF (2025)
1	Pain	72	859	11.93	Q1	5.5
2	Journal of Pain Research	47	282	6	Q2	2.5
3	European Journal of Pain	40	219	5.47	Q1	3.4
4	British Journal of Anesthesia	35	343	9.8	Q1	9.2
5	Regional Anesthesia and Pain Medicine	34	151	4.44	Q1	3.5
6	Clinical Journal of Pain	32	390	12.19	Q1	3.1
7	Journal of Pain	29	319	11	Q1	4
8	Current Opinion in Anesthesiology	29	140	4.83	Q2	2.1
9	Anesthesiology	28	410	14.64	Q1	9.1
10	Anesthesia and Analgesia	28	231	8.25	Q1	3.8
11	Pain Medicine	28	130	4.64	Q1	3

### 3.5 Keywords co-occurrence, clusters and bursts

**Figure 10:**
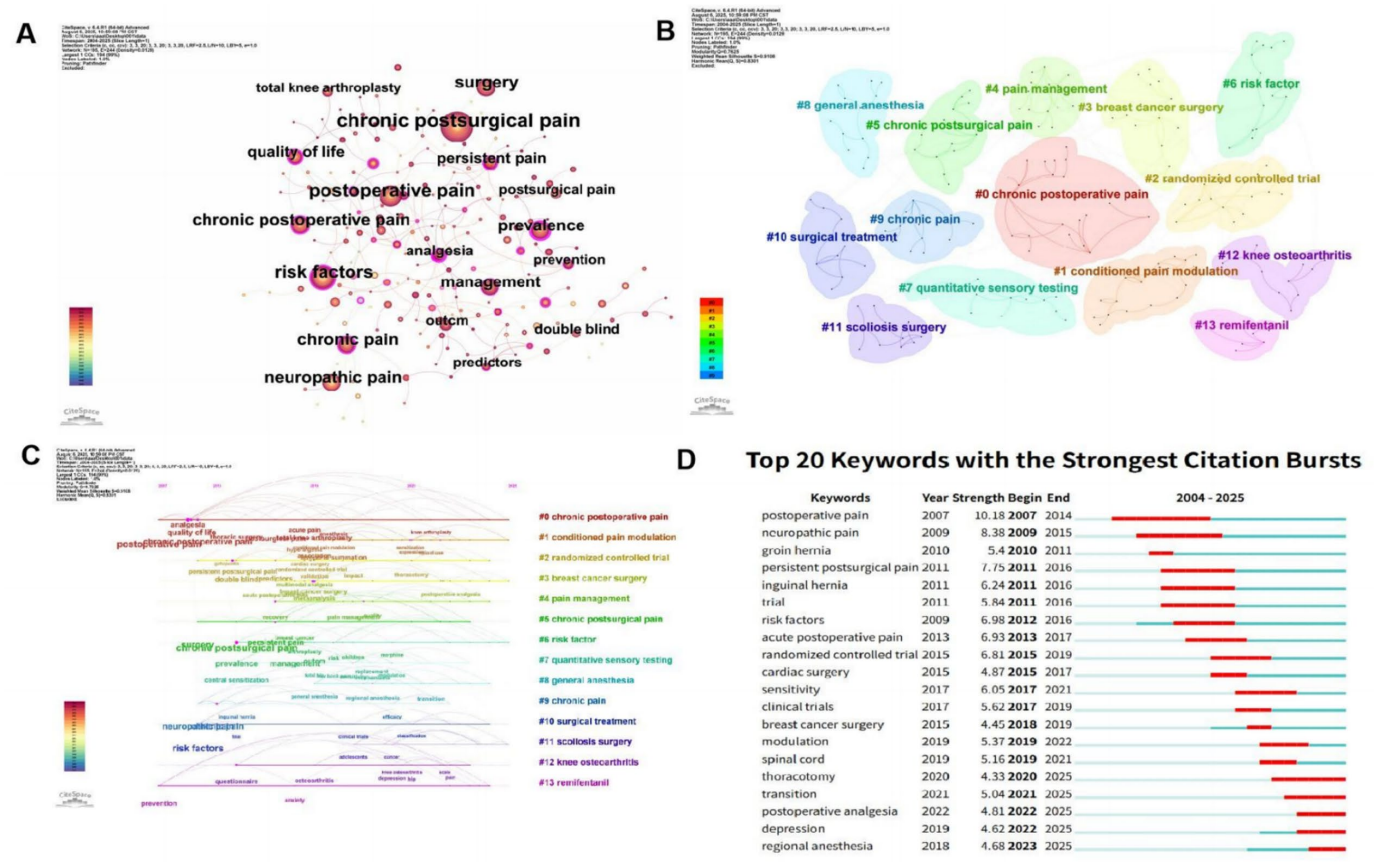
**(A)** Keyword network visualization in CPSP research. **(B)** Cluster distribution map of keywords in CPSP research. **(C)** Timeline chart of keyword clustering on CPSP. **(D)** Keyword burst chart of CPSP.

The co-occurrence map (Figure 10A) illustrates core keywords and their interrelationships within the field of CPSP research. Keywords such as “postoperative pain,” “chronic postoperative pain,” and “pain management” occupy central positions, indicating high research attention and influence in this field. Additionally, keywords like “quality of life” and “risk factors” exhibit strong correlations, indicating these factors are equally important in the study of CPSP. The cluster map (Figure 10B) illustrates the primary research directions and thematic clusters within the field of CPSP studies through color-coded regions. Fourteen major clusters are identified, with “Chronic Postoperative Pain” (#0) being the largest cluster, indicating its dominant position in research within this domain. Other significant clusters include “Pain Management” (#4), “Breast Cancer Surgery” (#3), and “Risk Factors” (#6), reflecting the diversity and complexity of CPSP research. The timeline diagram (Figure 10C) illustrates the evolution of key terms in CPSP research over time. Keywords such as “postoperative pain” and “pain management” began appearing around 2007 and remained consistently prominent in subsequent years. Additionally, keywords like “neuropathic pain” and “risk factors” started to emerge around 2011, indicating these topics are gaining increasing importance in research within this field. Through timeline analysis, research hotspots and development trends in the field of CPSP research can be clearly identified. The burst map (Figure 10D) displays the 20 keywords with the strongest burst intensity in the field of CPSP research, along with their burst timing. It is observed that “postoperative pain” exhibited the highest emergence intensity between 2007 and 2014, demonstrating its significant influence in research within this field. Other keywords with strong emergence intensity include “neuropathic pain,” “groin hernia,” and “persistent postoperative pain,” which exhibited notable research activity during different periods. This is not difficult to explain. Nerve trauma in surgical procedures like groin hernia repair primarily involves blunt peripheral axonal injury, including compression, stretching, perineural inflammation, entrapment, and scar formation, accompanied by entrapment of sensory fibers and/or neuroma formation. Regional anesthesia techniques may also damage peripheral nerves, leading to neuropathic pain (24, 25). Generally, partial injury to sensory axons during surgery leads to spontaneous activity and a lowered activation threshold, while also increasing responsiveness to normal stimuli. Hyperesthesia can be attributed to heightened sensitivity in uninterrupted but injured axons, resulting in increased abnormal sodium channel density that predisposes them to spontaneous ectopic discharge. Iatrogenically injured nerves may develop ectopic pacemakers at various sites along their length. Additionally, altered axonal receptor expression may increase sensitivity to algogenic substances, potentially eliciting responses to normally non-stimulating agents (7). Surgical-induced inflammatory responses can alter gene expression in dorsal root ganglia, increasing peripheral receptor synthesis and sensitizing nociceptors.

## 4 Discussion

CPSP, as a postoperative complication, significantly exacerbates the economic burden on individuals, families, and even the nation. Currently, treatment options for CPSP are limited, resulting in suboptimal clinical outcomes. Therefore, to further understand the temporal and spatial distribution of CPSP research, its primary contributors, and central publications, while identifying current research status and future hotspots and frontiers, we employed Citespace 6.2R4 Advanced, VOSviewer 1.6.19, and Bibliometrix 4.1.3 software. This analysis is based on data extracted from Web of Science database and Scopus database covering the period from 2004 to 2025. The upward trend in annual publication volume highlights the substantial research potential of CPSP, indicating a burgeoning field ripe for further exploration.

### 4.1 General information

From 2004 to 2025, the annual number of publications showed a clear upward trend. Particularly after 2018, the number of additional papers published each year exceeded 20, reflecting the field’s active research and technological advancements. Among all publications, the ratio of articles (950) to reviews (261) is approximately 3.6:1. This indicates that empirical research dominates studies on CPSP, while review articles remain relatively scarce. This may suggest the field is still in a phase of rapid development, requiring more foundational research to support theoretical frameworks. These findings underscore the growing recognition of CPSP as a clinical issue of widespread existence, significantly impacting patients’ quality of life. Advances in medical technology, particularly emerging therapies and techniques in pain management, have facilitated increased research activity. Future efforts should encourage interdisciplinary collaboration—such as between neuroscience, psychology, and pharmacology—to comprehensively understand the mechanisms of CPSP and develop more effective interventions.

The Nightingale rose diagram and chord diagram reveal that the United States leads in scientific output, followed by China, Canada, Denmark, and others. This indicates America’s dominance in CPSP research, likely attributable to its robust research infrastructure, ample funding, and extensive international collaboration networks. Although China ranks second in total output, it has a notably low number of renowned authors and universities in this field, suggesting it is currently in a phase of quantitative expansion and will need to enhance research quality in the future. Canada also performs exceptionally well, with frequent international collaborations reflecting its “quality-first” strategic positioning. Denmark, despite its small population, plays a leading role in this research domain, demonstrating high efficiency. Collectively, Denmark’s efficiency, the United States’ scale, Canada’s hub function, and China’s catch-up efforts shape the global landscape of CPSP research. Future policies should encourage efficiency-oriented collaborative models (such as Denmark’s specialty centers) rather than simple quantitative expansion. The diagram highlights institutions such as the University of Toronto, Aalborg University, and McGill University as key nodes within the collaboration network, indicating their significant influence and active engagement in the field of CPSP research. Future efforts should encourage broader collaboration among nations and institutions to facilitate knowledge exchange and deepen research.

When analyzing bibliometric studies on CPSP in the field of anesthesia, the collaboration network diagram and Lotka diagram generated by VOSviewer software, combined with the list of highly productive authors in Table 2, reveal that these authors exert significant academic influence in this domain. Not only do they publish extensively, but they also form core research teams within the field through collaborative networks. These teams may concentrate on current research hotspots and enhance publication efficiency through cooperation. This collaborative model not only facilitates knowledge exchange and innovation but also significantly influences the research direction and allocation of academic resources across the entire field. However, such concentrated resource allocation may impact the research of other authors. Therefore, future research recommendations should encourage broader collaboration, support diversity in research topics and teams, and focus on the growth of emerging authors to drive further development and innovation within the field.

Through a bibliometric analysis of journals in the field of anesthesiology, we found that journals in Bradford’s Zone 1, such as PAIN and JOURNAL OF PAIN RESEARCH, occupy a central position in terms of scientific output and citation frequency. These journals not only have high publication volumes, numerous citations, and high average citation counts, but also relatively high impact factors, demonstrating significant academic influence within the field. Collaboration network diagrams generated using VOSviewer software further reveal these journals’ pivotal role in constructing academic exchange and collaboration networks. Positioned at the network’s core, they indicate frequent collaborative relationships and high influence. Such distribution and network structures positively impact the guidance of research directions, enhancement of academic influence, and formation of interdisciplinary collaboration networks within anesthesiology. To advance the field, future research should continue strengthening interdisciplinary collaboration, improving research quality, and facilitating knowledge dissemination. These efforts will further elevate the academic influence and research depth within the field of anesthesiology.

### 4.2 Hotspots and frontiers

Keyword analysis helps identify the frontiers and focal points within a research field. In this study a comprehensive keyword analysis was conducted to describe major trends and temporal shifts in the field of CPSP. Keywords identified through co-occurrence network analysis (Figure 10A) include “postoperative pain,” “chronic postoperative pain,” “pain management,” “quality of life,” and “risk factors.” These keywords primarily address the prevention management and impact on patient quality of life associated with CPSP indicating these topics are currently at the forefront of research in this field. Using VOSviewer software for visual mapping CPSP research was categorized into 14 major directions (Figure 10B). These clusters not only highlight the diversity of research but also suggest potential interconnections between different research pathways. For example, this figure displays the research theme cluster for CPSP (#0). First, CPSP is closely related to pain management (#4), which involves assessing pain and developing treatment strategies. A key aspect of pain management is conditioned pain modulation (#1), which aids in understanding the physiological mechanisms of pain. To better quantify pain, researchers conduct quantitative sensory testing (#7) to assess pain intensity and characteristics. Among specific surgical procedures, breast cancer surgery (#3) and scoliosis surgery (#11) are common sources of CPSP. For these surgeries, risk factors (#6) such as surgical techniques and patient health status may influence postoperative pain occurrence. Additionally, randomized controlled trials (#2) serve as a crucial study design for evaluating the efficacy of different treatment approaches on CPSP. Treatment modalities encompass not only pain management but also specialized therapies for chronic pain (#9) and CPSP (#5), alongside surgical interventions (#10) such as joint replacement. Knee osteoarthritis (#12) may require specific treatment strategies. Remifentanil (#13) may play a role in pain management as a pharmaceutical agent. Finally, the use of general anesthesia (#8) during surgery may also influence the occurrence and duration of postoperative pain. Collectively, these topics form a comprehensive understanding of CPSP research, spanning fundamental mechanisms to clinical interventions across multiple dimensions. Figure 10C’s timeline diagram clearly illustrates the evolution of core terminology within the field of CPSP research. The figure shows how keywords such as “postoperative pain,” “pain management,” “neuropathic pain,” and “risk factors” emerged and shifted in prominence across different time periods. This information helps researchers understand research hotspots and development trends in the field. Figure 10D displays the emergence timing and intensity of the 20 most prominent keywords in CPSP research. Data indicates that “postoperative pain” exhibited its highest research intensity between 2007 and 2014, reflecting its significant influence in this field. Other notable keywords include “neuropathic pain,” “inguinal hernia,” and “persistent postoperative pain,” which exhibit active research trends across different time periods. By analyzing the burst distribution diagram, we can accurately identify core keywords and academic hotspots within this research domain.

### 4.3 Hot topics and future prospects in CPSP research

The discussion will primarily focus on the following areas of research in CPSP: risk factors, the mechanisms underlying its development, and strategies for managing postoperative pain.

#### 4.3.1 Risk factor

**Figure 11:**
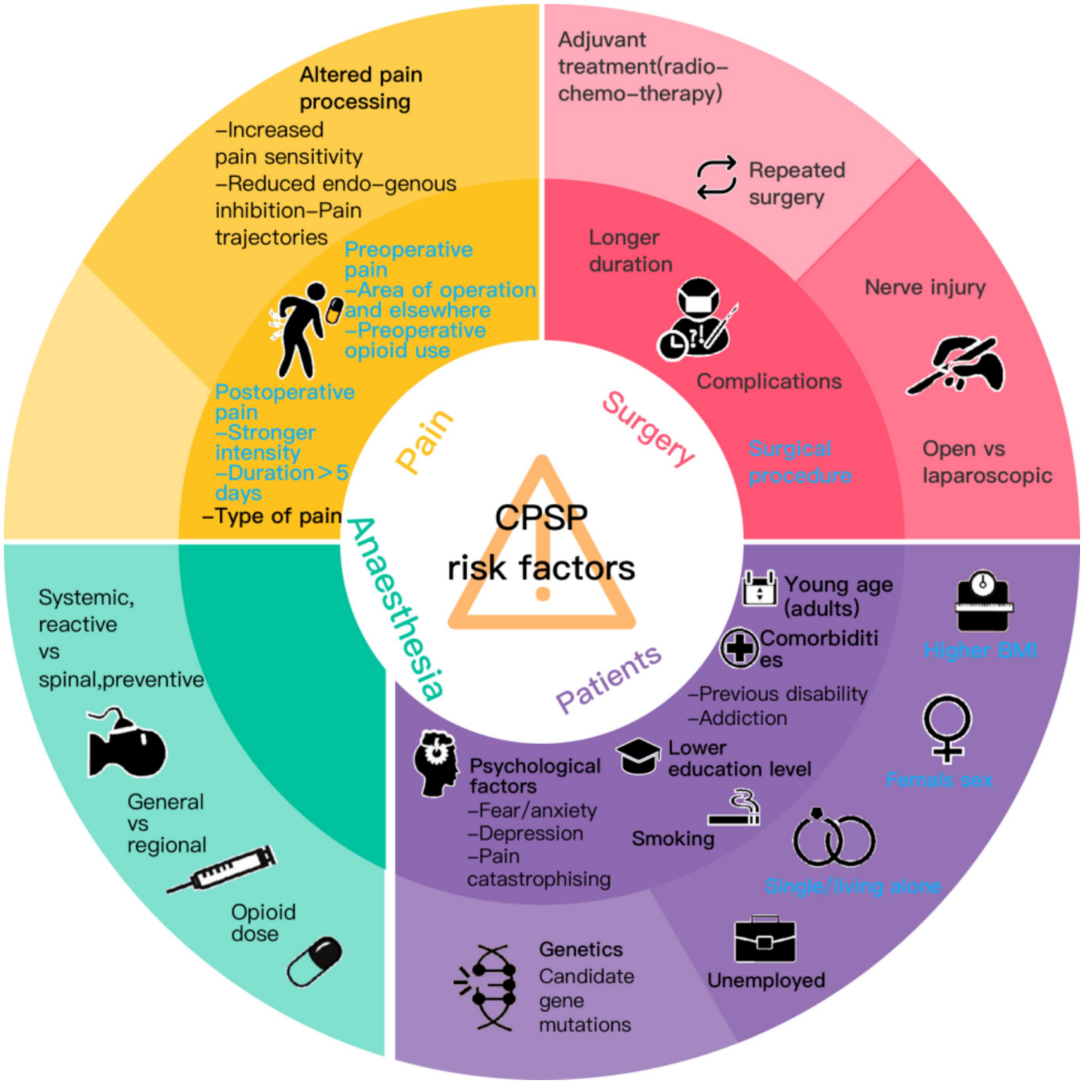
Risk factors for CPSP.

Although numerous risk factors for CPSP have been identified, no single dominant risk factor has been established (26). These risk factors can be categorized into four major groups: surgery-related, anesthesia-related, pain-related, and patient-related (Figure 11). They may significantly increase the overall risk through synergistic effects. Currently, assessment tools and risk stratification algorithms are in the development or early application stages, but limitations remain (27). Clinically, tools such as the Hospital Anxiety and Depression Scale, Pain Anxiety Symptom Scale, and Beck Depression Inventory are commonly used (28, 29), yet no validated and widely accepted assessment tool specifically targeting CPSP exists. Future efforts should integrate modifiable and non-modifiable risk factors to develop nomograms or risk scoring systems tailored to specific surgical types. The emphasis lies in dynamic risk assessment—continuous monitoring spanning the preoperative, intraoperative, and acute postoperative periods. Furthermore, both genetics and epigenetics play significant roles, though relevant literature and research remain relatively scarce at present. Future efforts should focus on identifying novel CPSP susceptibility gene loci through large-scale cohort studies, while simultaneously tracking the temporal changes in specific gene methylation and histone modifications during the perioperative period to uncover the molecular switches governing the transition from acute to chronic pain. Notably, many risk factors are modifiable, though evidence remains insufficient in some cases (30). Due to the requirement for longitudinal patient follow-up and self-reported pain experiences via questionnaires, this research methodology inherently carries a risk of bias, resulting in certain inherent limitations in CPSP studies. Future research should shift focus from identifying population-level risk factors to individualized risk prediction, from correlational studies to elucidating causal mechanisms, and from single interventions to multidimensional integrated prevention systems. Particularly crucial is the establishment of an internationally standardized CPSP registry database employing uniform diagnostic criteria (e.g., ICD-11 definitions) and long-term follow-up protocols to address the fundamental issues of current research fragmentation and high heterogeneity.

#### 4.3.2 Comparison with specialty- and population-specific bibliometric studies

While the present study provides a comprehensive overview of CPSP research across all surgical domains, several bibliometric analyses have focused on specific surgical specialties or patient populations, offering complementary perspectives that warrant comparative discussion.

##### 4.3.2.1 Specialty-specific bibliometric studies

Recent bibliometric investigations have examined CPSP within individual surgical domains. For instance, Shanthanna et al.’s bibliometric analysis of thoracic surgery identified intercostal nerve injury as a dominant mechanism, aligning with our finding that “thoracotomy” exhibited strong keyword burst intensity between 2011 and 2016 (Figure 10D) (31). However, our broader analysis reveals that thoracic surgery represents only one of 14 major research clusters (Figure 10B), with breast surgery (#3) and general anesthesia (#8) showing comparable centrality, suggesting that specialty-specific studies may overemphasize domain-specific mechanisms while undervaluing cross-cutting themes such as central sensitization. Similarly, bibliometric reviews in obstetric surgery have highlighted the unique contribution of hormonal factors to CPSP risk, which our general framework consolidates under the broader “gender differences” category (32–34). The present study’s inclusive approach demonstrates that gender as a risk factor spans multiple specialties (risk factor #6 cluster), whereas specialty-focused analyses might miss this trans-surgical pattern.

##### 4.3.2.2 Population-specific bibliometric research

Population-targeted bibliometric studies have yielded distinct insights. Pediatric CPSP bibliometric analyses consistently identify “age” as a protective factor, contrasting with our finding that younger age (<55 years) increases CPSP risk in adult populations (35, 36). This discrepancy underscores the necessity of age-stratified research—a gap our study addresses by revealing age as a contextual risk factor whose effect directionality depends on developmental stage, a nuance often lost in age-restricted analyses. Geriatric-focused bibliometric reviews emphasize frailty and comorbidity burden (37, 38), which our analysis integrates into the broader “risk factors” cluster (Figure 10B).

##### 4.3.2.3 Novelty and added value of the present study

The uniqueness of our work lies in three aspects: First, temporal comprehensiveness: Our 21-year analysis (2004–2025) encompasses the entire modern CPSP research era, whereas specialty-specific studies typically examine shorter periods (e.g., 10-year windows), potentially missing long-term paradigm shifts such as the rise of “neuropathic pain” research after 2011 (Figure 10C). Second, methodological integration: We combined Bradford’s Law, Lotka’s Law, and burst detection to reveal both structural (journal/author hierarchies) and dynamic (emerging frontiers) aspects simultaneously—a methodological breadth absent in narrower studies that often focus solely on keyword co-occurrence. Third, cross-specialty pattern recognition: By mapping 14 distinct clusters, we identified trans-surgical mechanisms (e.g., central sensitization, glial activation) that specialty-specific analyses treat as peripheral. Our network analysis (Figure 10A) positions “pain management” as a central node bridging all surgical domains, a metastructure invisible in single-specialty studies.

In essence, while specialty-and population-specific bibliometric studies provide depth, our comprehensive analysis provides the transversal framework necessary for identifying universal CPSP mechanisms and guiding resource allocation across the entire surgical landscape. This panoramic view is essential for developing standardized preventive strategies that can be adapted to specialty-specific contexts.

#### 4.3.3 Unresolved questions in the research of CPSP mechanisms

The chronicization process of CPSP is complex, with its underlying pathophysiology involving mechanisms such as peripheral sensitization, central sensitization, and glial cell activation. Although the molecular mechanisms of CPSP have been described in numerous studies (39–42), the following issues remain unresolved and require urgent attention.

##### 4.3.3.1 Peripheral sensitization

While peripheral sensitization serves as the initial phase of CPSP, the specific signals triggering irreversible central sensitization remain unclear (43, 44). Moreover, most current research relies on animal studies (45, 46), and the absence of human biomarkers for peripheral sensitization prevents the provision of guidance for individualized pain management. Future research should integrate optogenetics, spatial genomics, and metabolomics to reshape our understanding of peripheral mechanisms in CPSP. This interdisciplinary approach will lay the groundwork for developing non-opioid therapies that target the source of sensitization.

##### 4.3.3.2 Central sensitization

Although CPSP shares numerous common mechanisms with other types of pain, it remains a distinct form of chronic pain with unique characteristics. However, existing research has largely focused on the shared mechanisms across various pain conditions rather than the specific manifestations of CPSP. Furthermore, while animal models have demonstrated that central sensitization involves AMPA receptor phosphorylation and brain-derived neurotrophic factor (BDNF), the unavailability of human postoperative spinal cord samples limits mechanism validation to correlational rather than causal levels (47, 48). Although existing studies suggest genetic polymorphisms influence CPSP risk (49, 50), they fail to elucidate how specific genotypes shape individual differences in central sensitization thresholds, thereby preventing risk stratification. Future studies should synchronously compare spinal cord single-cell transcriptomes, phosphoproteomes, and epigenomes across postoperative incision models, neuropathic pain models, and inflammatory pain models to identify CPSP-specific differentially expressed genes and shared pathways. Mapping dynamic molecular trajectories across the postoperative acute phase (24 h), subacute phase (7 days), and chronic conversion phase (3 months) will pinpoint CPSP-specific molecules emerging exclusively during chronic conversion. Conduct an international multicenter CPSP cohort study (including surgical type, analgesic regimen, psychological scales, and immune markers) with whole-genome sequencing to identify functional variants associated with central sensitization thresholds. Simultaneously establish an international CPSP research consortium to share raw sequencing data, imaging data, and clinical outcomes from animal models and clinical cohorts. Utilize federated learning to train AI models, enabling big data causal inference while protecting privacy.

##### 4.3.3.3 Glial activation

In chronic pain models, various proinflammatory factors such as cytokines including tumor necrosis factor-alpha (TNF-*α*), interleukin-beta-1 (IL-β1), and interleukin-6 (IL-β6), as well as multiple chemokines, can trigger microglial activation (51, 52). Current rodent incision models can only simulate acute pain lasting from hours to a week, whereas human CPSP persists for months or even years, making it difficult to replicate the regulatory effects of psychosocial factors (such as pain catastrophizing and preoperative anxiety/depression) on glial cells. Furthermore, the temporal dynamics of glial cell activation during the early postoperative phase (within 24 h) versus the chronic phase (after 3 months) lack systematic tracking, making it difficult to identify the optimal intervention window. Although potential biomarkers such as the neutrophil-to-lymphocyte ratio and serum miRNAs have been identified, their specificity and reproducibility remain insufficient for personalized risk assessment (53, 54). Future research should elucidate how stress-glucocorticoids-HPA axis reshape microglial function (e.g., p38 MAPK phosphorylation) and explore the regulatory effects of antidepressant therapy on glial cells. Additionally, cell atlases of the spinal cord, dorsal root ganglia, and brain regions at different postoperative time points should be mapped to reveal subtype-specific transcriptional features of glial cells and their interaction networks with neurons. More importantly, establishing a CPSP risk prediction model based on serum miRNA and cytokine profiles (e.g., IL-6/IL-10 ratio) is essential to guide perioperative analgesia strategies (e.g., reducing opioid dosage) (51, 55).

#### 4.3.4 Management

Preemptive analgesia refers to the administration of analgesics before the initial surgical incision or during the perioperative period (56). Although no consensus exists on the optimal preventive strategy (57), multiple approaches have been proposed. To prevent central pain syndrome, primary afferent nerve stimulation must be blocked or inhibited. Preventive measures include local anesthetic infiltration, multimodal analgesia, or monotherapy with the lowest effective dose of opioids (58). For cancer patients, scientifically designed multimodal analgesia protocols can reduce the risk of CPSP (36, 59). However, the optimal multimodal analgesia regimen remains unclear, requiring consideration of factors such as the type of surgery, patient variability, and drug interactions. Research on the most effective strategies for preventing CPSP remains insufficient.

**Figure 12:**
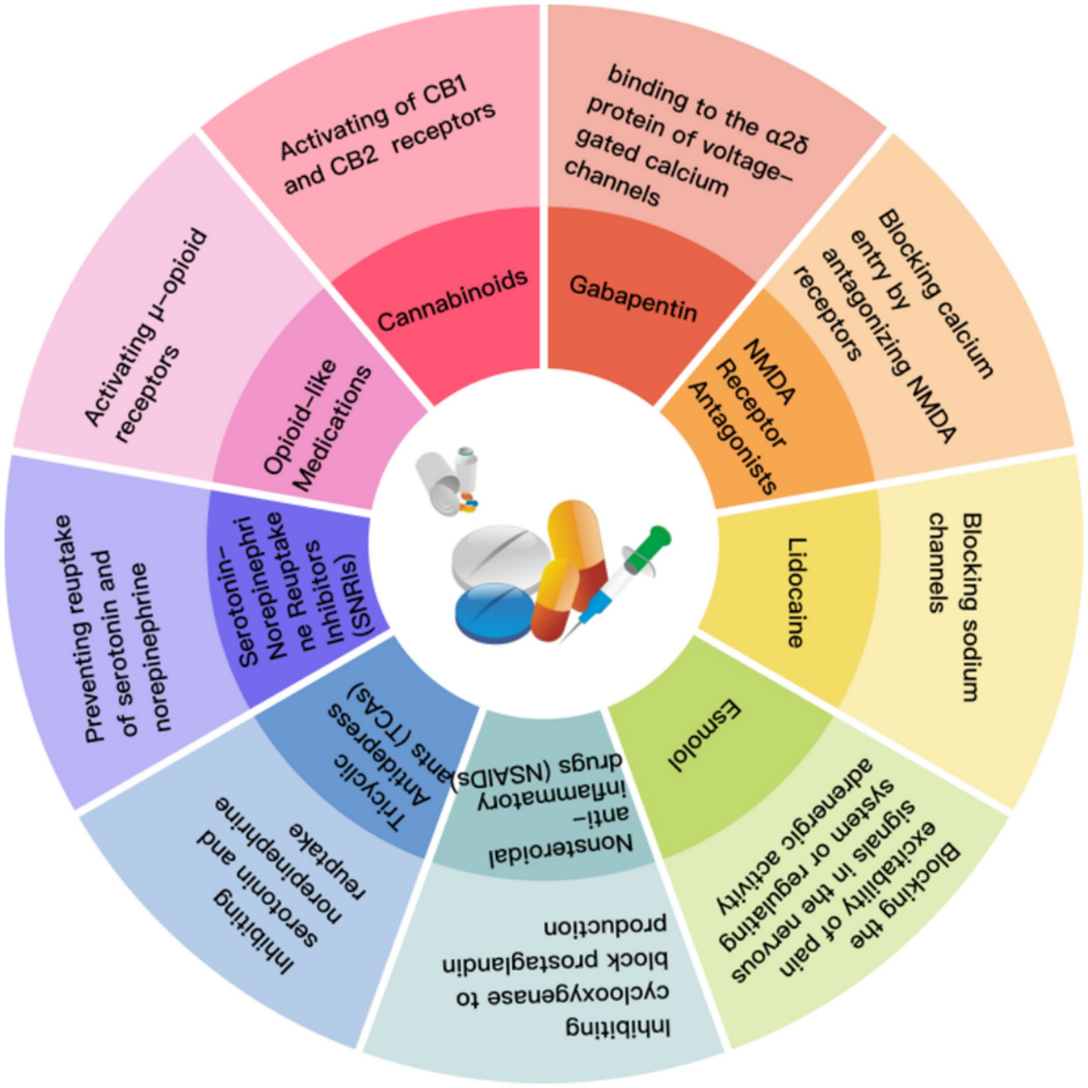
Pharmacological diagram of various analgesics and co-analgesics.

Preemptive analgesic medications include gabapentin, ketamine, lidocaine, esmolol, dextromethorphan, and dexmedetomidine (Figure 12) (60–62). Intravenous lidocaine administered perioperatively may confer postoperative analgesic benefits by inhibiting afferent neural pathways to alleviate or prevent neuropathic pain (63). Other local anesthetics may exhibit similar effects (64). Although ketamine has garnered attention, evidence supporting its efficacy in reducing postoperative pain is limited or of low quality (60). Esmolol reduces postoperative opioid requirements and postoperative care unit opioid consumption while alleviating acute pain following mastectomy (65). However, its use is contraindicated in patients with heart failure or severe cardiac arrhythmias (60). Esmolol reduces intraoperative opioid requirements and postoperative intensive care unit opioid consumption, and alleviates acute pain after mastectomy (65), though its effect on continuous pain scores remains unclear (66).

While studies have evaluated analgesics for relieving acute postoperative pain, their potential to prevent or eliminate PPS has not been fully validated in large-scale, long-term randomized clinical trials (61). It is widely recognized that implementing prophylactic analgesia or aggressive pain control immediately postoperatively may help reduce the risk of CPSP. A study involving 174 surgical patients demonstrated that the degree of acute pain relief was a more effective predictor of avoiding CPSP at 6 months than the intensity of postoperative pain alone (67). Multimodal postoperative analgesia protocols are considered to provide superior perioperative and postoperative pain management. Conventional approaches include N-methyl-D-aspartate (NMDA) receptor antagonists, nonsteroidal anti-inflammatory drugs (NSAIDs), acetaminophen, intravenous lidocaine, peripheral nerve blocks, and epidural blocks (Figure 12) (68). However, the challenge of multimodal analgesia lies in tailoring protocols to individual patient needs, comorbidities, and surgical procedures. Furthermore, multimodal approaches can extend to non-pharmacological interventions such as psychological counseling, physical therapy, and exercise therapy, potentially requiring multidisciplinary pain teams to assess suitable candidates. Although opioids remain difficult to eliminate from postoperative pain management regimens in the short term, the medical community is increasingly recognizing that rational opioid use combined with exploration of alternative analgesic options represents the current optimal management strategy.

Most risk factors for chronic pain syndromes are nonmodifiable and may be triggered by the necessity of surgery. The optimal strategy involves alleviating acute postoperative pain while maintaining continuous patient monitoring.

### 4.4 Limitations

Undeniably, this study has several limitations. Initially, our data was sourced solely from the WoSCC database and Scopus database, which exclusively includes English-language publications in the formats of Articles and Reviews. This may result in incomplete data and analytical findings, potentially introducing bias in the literature database. Furthermore, significant publications in other languages remain excluded from this study, potentially missing diverse perspectives and insights from non-English sources. This may impact the comprehensive understanding of global research trends, introducing language bias. Nevertheless, given the minimal proportion of non-English articles, the trends identified in our research remain a valuable reference. Second, our search was completed on August 5, 2025, potentially overlooking some recently updated papers during the study period. Furthermore, some high-quality studies published recently may have been overlooked due to low citation counts. At the same time, search results may vary due to differences in the scope of databases purchased by various organizations. Finally, this bibliometric analysis primarily employs absolute publication volume as its core metric without standardizing for variables such as population size, GDP, healthcare expenditure, or research funding across nations. While this methodology provides an intuitive reflection of the global research landscape, it does introduce potential biases—larger nations naturally produce more data due to their scale and resources.

## 5 Conclusion

This study discusses research hotspots and frontier issues in CPSP through a bibliometric analysis and review based on the Web of Science database and Scopus database. Results indicate an upward trend in research within this field, with countries such as the United States, China, Canada, and Denmark demonstrating prominent contributions. Several renowned universities and medical institutions have played significant roles in this research. Furthermore, the study delves into risk factors, pathogenesis, and management strategies for CPSP, emphasizing the importance of multidisciplinary collaboration. It suggests that future research should focus on elucidating the mechanisms underlying CPSP in peripheral sensitization, central sensitization, and glial cell activation. Preventive analgesia, multimodal pain management, and non-pharmacological interventions are considered beneficial for reducing CPSP risk, though no universally optimal approach has yet been established. Future efforts should focus on developing more effective interventions based on a comprehensive understanding of the underlying mechanisms, aiming to reduce the incidence of CPSP.

## Data Availability

The original contributions presented in the study are included in the article/Supplementary material, further inquiries can be directed to the corresponding author.

## Footnotes

1
https://bibliometric.com/
